# Supplementary material for: Spatio-spectral classification of hyperspectral images for brain cancer detection during surgical operations
Source: PLoS One. 2018 Mar 19;13(3):e0193721. doi: 10.1371/journal.pone.0193721 (PMC5858847; doi:10.1371/journal.pone.0193721)
Supplement: S1 Table — (DOCX) [file pone.0193721.s001.docx]

|  |  | **Predicted Results (#pixels)** | | | | |  |  |
| --- | --- | --- | --- | --- | --- | --- | --- | --- |
| **Patient ID** | **Ground Truth Data (#pixels)** | **Normal Tissue** | **Tumor Tissue** | **Blood Vessel** | **Background** | **Total** | **Sensitivity (%)** | **FNR (%)** |
| **1** | **Normal Tissue** | 2295 | 0 | 0 | 0 | 2295 | 100.00 | 0.00 |
|  | **Tumor Tissue** | 0 | 1201 | 20 | 0 | 1221 | 98.36 | 1.64 |
|  | **Blood Vessel** | 0 | 16 | 1315 | 0 | 1331 | 98.80 | 1.20 |
|  | **Background** | 0 | 0 | 0 | 630 | 630 | 100.00 | 0.00 |
|  | **Total** | 2295 | 1217 | 1335 | 630 | 5477 |  | |
|  | **Specificity (%)** | 100.00 | 99.62 | 99.52 | 100.00 |  | **Overall Accuracy (%)** | **99.34** |
|  | **FPR (%)** | 0.00 | 0.38 | 0.48 | 0.00 |  |  |  |
|  | | | | | | | | |
|  | | **Predicted Results (#pixels)** | | | | |  | |
| **Patient ID** | **Ground Truth Data (#pixels)** | **Normal Tissue** | **Tumor Tissue** | **Blood Vessel** | **Background** | **Total** | **Sensitivity (%)** | **FNR (%)** |
| **2** | **Normal Tissue** | 4502 | 0 | 14 | 0 | 4516 | 99.69 | 0.31 |
|  | **Tumor Tissue** | 0 | 853 | 2 | 0 | 855 | 99.77 | 0.23 |
|  | **Blood Vessel** | 5 | 0 | 8692 | 0 | 8697 | 99.94 | 0.06 |
|  | **Background** | 5 | 2 | 0 | 1678 | 1685 | 99.58 | 0.42 |
|  | **Total** | 4512 | 855 | 8708 | 1678 | 15753 |  | |
|  | **Specificity (%)** | 99.91 | 99.99 | 99.77 | 100.00 |  | **Overall Accuracy (%)** | **99.82** |
|  | **FPR (%)** | 0.09 | 0.01 | 0.23 | 0.00 |  |  |  |
|  | | | | | | | | |
|  | | **Predicted Results (#pixels)** | | | | |  | |
| **Patient ID** | **Ground Truth Data (#pixels)** | **Normal Tissue** | **Tumor Tissue** | **Blood Vessel** | **Background** | **Total** | **Sensitivity (%)** | **FNR (%)** |
| **3** | **Normal Tissue** | 1250 | 0 | 1 | 0 | 1251 | 99.92 | 0.08 |
|  | **Tumor Tissue** | 0 | 2046 | 0 | 0 | 2046 | 100.00 | 0.00 |
|  | **Blood Vessel** | 0 | 0 | 4089 | 0 | 4089 | 100.00 | 0.00 |
|  | **Background** | 0 | 1 | 0 | 695 | 696 | 99.86 | 0.14 |
|  | **Total** | 1250 | 2047 | 4090 | 695 | 8082 |  |  |
|  | **Specificity (%)** | 100.00 | 99.98 | 99.97 | 100.00 |  | **Overall Accuracy (%)** | **99.98** |
|  | **FPR (%)** | 0.00 | 0.02 | 0.03 | 0.00 |  |  |  |
|  | | | | | | | | |
|  | | **Predicted Results (#pixels)** | | | | |  | |
| **Patient ID** | **Ground Truth Data (#pixels)** | **Normal Tissue** | **Tumor Tissue** | **Blood Vessel** | **Background** | **Total** | **Sensitivity (%)** | **FNR (%)** |
| **4** | **Normal Tissue** | 1823 | 0 | 19 | 0 | 1842 | 98.97 | 1.03 |
|  | **Tumor Tissue** | 0 | 3655 | 0 | 0 | 3655 | 100.00 | 0.00 |
|  | **Blood Vessel** | 31 | 0 | 1482 | 0 | 1513 | 97.95 | 2.05 |
|  | **Background** | 0 | 0 | 0 | 2625 | 2625 | 100.00 | 0.00 |
|  | **Total** | 1854 | 3655 | 1501 | 2625 | 9635 |  | |
|  | **Specificity (%)** | 99.60 | 100.00 | 99.77 | 100.00 |  | **Overall Accuracy (%)** | **99.48** |
|  | **FPR (%)** | 0.40 | 0.00 | 0.23 | 0.00 |  |  |  |
|  | | | | | | | | |
|  | | **Predicted Results (#pixels)** | | | | |  | |
| **Patient ID** | **Ground Truth Data (#pixels)** | **Normal Tissue** | **Tumor Tissue** | **Blood Vessel** | **Background** | **Total** | **Sensitivity (%)** | **FNR (%)** |
| **5** | **Normal Tissue** | 977 | 0 | 0 | 0 | 977 | 100.00 | 0.00 |
|  | **Tumor Tissue** | 0 | 1221 | 0 | 0 | 1221 | 100.00 | 0.00 |
|  | **Blood Vessel** | 0 | 0 | 907 | 0 | 907 | 100.00 | 0.00 |
|  | **Background** | 0 | 0 | 0 | 2503 | 2503 | 100.00 | 0.00 |
|  | **Total** | 977 | 1221 | 907 | 2503 | 5608 |  | |
|  | **Specificity (%)** | 100.00 | 100.00 | 100.00 | 100.00 |  | **Overall Accuracy (%)** | **100.00** |
|  | **FPR (%)** | 0.00 | 0.00 | 0.00 | 0.00 |  |  |  |

**S1 Table. Confusion matrix results of the SVM supervised classification with linear kernel applying the 10-fold cross validation method to each patient.**
