# Supplementary material for: Spatio-spectral classification of hyperspectral images for brain cancer detection during surgical operations
Source: PLoS One. 2018 Mar 19;13(3):e0193721. doi: 10.1371/journal.pone.0193721 (PMC5858847; doi:10.1371/journal.pone.0193721)
Supplement: S2 Table — (DOCX) [file pone.0193721.s002.docx]

|  |  | **Predicted Results (#pixels)** | | | | |  |  |
| --- | --- | --- | --- | --- | --- | --- | --- | --- |
| **Patient ID** | **Ground Truth Data (#pixels)** | **Normal Tissue** | **Tumor Tissue** | **Blood Vessel** | **Background** | **Total** | **Sensitivity (%)** | **FNR (%)** |
| **1** | **Normal Tissue** | 2291 | 4 | 0 | 0 | 2295 | 99.83 | 0.17 |
|  | **Tumor Tissue** | 0 | 1220 | 1 | 0 | 1221 | 99.92 | 0.08 |
|  | **Blood Vessel** | 10 | 682 | 639 | 0 | 1331 | 48.01 | 51.99 |
|  | **Background** | 0 | 0 | 0 | 630 | 630 | 100.00 | 0.00 |
|  | **Total** | 2301 | 1906 | 640 | 630 | 5477 |  | |
|  | **Specificity (%)** | 99.60 | 83.84 | 99.98 | 100.00 |  | **Overall Accuracy (%)** | **87.27** |
|  | **FPR (%)** | 0.40 | 16.16 | 0.02 | 0.00 |  |  |  |
|  | | | | | | | | |
|  | | **Predicted Results (#pixels)** | | | | |  | |
| **Patient ID** | **Ground Truth Data (#pixels)** | **Normal Tissue** | **Tumor Tissue** | **Blood Vessel** | **Background** | **Total** | **Sensitivity (%)** | **FNR (%)** |
| **2** | **Normal Tissue** | 4484 | 14 | 18 | 0 | 4516 | 99.29 | 0.71 |
|  | **Tumor Tissue** | 50 | 554 | 251 | 0 | 855 | 64.80 | 35.20 |
|  | **Blood Vessel** | 12 | 8 | 8677 | 0 | 8697 | 99.77 | 0.23 |
|  | **Background** | 25 | 5 | 0 | 1655 | 1685 | 98.22 | 1.78 |
|  | **Total** | 4571 | 581 | 8946 | 1655 | 15753 |  | |
|  | **Specificity (%)** | 99.21 | 99.82 | 96.14 | 100.00 |  | **Overall Accuracy (%)** | **97.57** |
|  | **FPR (%)** | 0.79 | 0.18 | 3.86 | 0.00 |  |  |  |
|  | | | | | | | | |
|  | | **Predicted Results (#pixels)** | | | | |  | |
| **Patient ID** | **Ground Truth Data (#pixels)** | **Normal Tissue** | **Tumor Tissue** | **Blood Vessel** | **Background** | **Total** | **Sensitivity (%)** | **FNR (%)** |
| **3** | **Normal Tissue** | 1232 | 7 | 12 | 0 | 1251 | 98.48 | 1.52 |
|  | **Tumor Tissue** | 1 | 2045 | 0 | 0 | 2046 | 99.95 | 0.05 |
|  | **Blood Vessel** | 1 | 0 | 4088 | 0 | 4089 | 99.98 | 0.02 |
|  | **Background** | 0 | 21 | 1 | 674 | 696 | 96.84 | 3.16 |
|  | **Total** | 1234 | 2073 | 4101 | 674 | 8082 |  |  |
|  | **Specificity (%)** | 99.97 | 99.54 | 99.67 | 100.00 |  | **Overall Accuracy (%)** | **99.47** |
|  | **FPR (%)** | 0.03 | 0.46 | 0.33 | 0.00 |  |  |  |
|  | | | | | | | | |
|  | | **Predicted Results (#pixels)** | | | | |  | |
| **Patient ID** | **Ground Truth Data (#pixels)** | **Normal Tissue** | **Tumor Tissue** | **Blood Vessel** | **Background** | **Total** | **Sensitivity (%)** | **FNR (%)** |
| **4** | **Normal Tissue** | 1823 | 1 | 18 | 0 | 1842 | 98.97 | 1.03 |
|  | **Tumor Tissue** | 0 | 3655 | 0 | 0 | 3655 | 100.00 | 0.00 |
|  | **Blood Vessel** | 356 | 0 | 1157 | 0 | 1513 | 76.47 | 23.53 |
|  | **Background** | 0 | 0 | 0 | 2625 | 2625 | 100.00 | 0.00 |
|  | **Total** | 2179 | 3656 | 1175 | 2625 | 9635 |  | |
|  | **Specificity (%)** | 95.43 | 99.98 | 99.78 | 100.00 |  | **Overall Accuracy (%)** | **96.11** |
|  | **FPR (%)** | 4.57 | 0.02 | 0.22 | 0.00 |  |  |  |
|  | | | | | | | | |
|  | | **Predicted Results (#pixels)** | | | | |  | |
| **Patient ID** | **Ground Truth Data (#pixels)** | **Normal Tissue** | **Tumor Tissue** | **Blood Vessel** | **Background** | **Total** | **Sensitivity (%)** | **FNR (%)** |
| **5** | **Normal Tissue** | 977 | 0 | 0 | 0 | 977 | 100.00 | 0.00 |
|  | **Tumor Tissue** | 0 | 1221 | 0 | 0 | 1221 | 100.00 | 0.00 |
|  | **Blood Vessel** | 111 | 0 | 727 | 69 | 907 | 80.15 | 19.85 |
|  | **Background** | 0 | 0 | 0 | 2503 | 2503 | 100.00 | 0.00 |
|  | **Total** | 1088 | 1221 | 727 | 2572 | 5608 |  | |
|  | **Specificity (%)** | 97.57 | 100.00 | 100.00 | 97.70 |  | **Overall Accuracy (%)** | **96.79** |
|  | **FPR (%)** | 2.43 | 0.00 | 0.00 | 2.30 |  |  |  |

**S2 Table. Confusion matrix results of the SVM supervised classification with polynomial kernel applying the 10-fold cross validation method to each patient.**
