# Supplementary material for: Spatio-spectral classification of hyperspectral images for brain cancer detection during surgical operations
Source: PLoS One. 2018 Mar 19;13(3):e0193721. doi: 10.1371/journal.pone.0193721 (PMC5858847; doi:10.1371/journal.pone.0193721)
Supplement: S3 Table — (DOCX) [file pone.0193721.s003.docx]

|  |  | **Predicted Results (#pixels)** | | | | |  |  |
| --- | --- | --- | --- | --- | --- | --- | --- | --- |
| **Patient ID** | **Ground Truth Data (#pixels)** | **Normal Tissue** | **Tumor Tissue** | **Blood Vessel** | **Background** | **Total** | **Sensitivity (%)** | **FNR (%)** |
| **1** | **Normal Tissue** | 2295 | 0 | 0 | 0 | 2295 | 100.00 | 0.00 |
|  | **Tumor Tissue** | 0 | 1221 | 0 | 0 | 1221 | 100.00 | 0.00 |
|  | **Blood Vessel** | 2 | 581 | 748 | 0 | 1331 | 56.20 | 43.80 |
|  | **Background** | 0 | 0 | 0 | 630 | 630 | 100.00 | 0.00 |
|  | **Total** | 2297 | 1802 | 748 | 630 | 5477 |  | |
|  | **Specificity (%)** | 99.92 | 86.34 | 100.00 | 100.00 |  | **Overall Accuracy (%)** | **89.36** |
|  | **FPR (%)** | 0.08 | 13.66 | 0.00 | 0.00 |  |  |  |
|  | | | | | | | | |
|  | | **Predicted Results (#pixels)** | | | | |  | |
| **Patient ID** | **Ground Truth Data (#pixels)** | **Normal Tissue** | **Tumor Tissue** | **Blood Vessel** | **Background** | **Total** | **Sensitivity (%)** | **FNR (%)** |
| **2** | **Normal Tissue** | 4497 | 0 | 19 | 0 | 4516 | 99.58 | 0.42 |
|  | **Tumor Tissue** | 52 | 744 | 59 | 0 | 855 | 87.02 | 12.98 |
|  | **Blood Vessel** | 5 | 4 | 8688 | 0 | 8697 | 99.90 | 0.10 |
|  | **Background** | 19 | 6 | 1 | 1659 | 1685 | 98.46 | 1.54 |
|  | **Total** | 4573 | 754 | 8767 | 1659 | 15753 |  | |
|  | **Specificity (%)** | 99.32 | 99.93 | 98.87 | 100.00 |  | **Overall Accuracy (%)** | **98.95** |
|  | **FPR (%)** | 0.68 | 0.07 | 1.13 | 0.00 |  |  |  |
|  | | | | | | | | |
|  | | **Predicted Results (#pixels)** | | | | |  | |
| **Patient ID** | **Ground Truth Data (#pixels)** | **Normal Tissue** | **Tumor Tissue** | **Blood Vessel** | **Background** | **Total** | **Sensitivity (%)** | **FNR (%)** |
| **3** | **Normal Tissue** | 1242 | 2 | 7 | 0 | 1251 | 99.28 | 0.72 |
|  | **Tumor Tissue** | 0 | 2046 | 0 | 0 | 2046 | 100.00 | 0.00 |
|  | **Blood Vessel** | 5 | 0 | 4084 | 0 | 4089 | 99.88 | 0.12 |
|  | **Background** | 0 | 22 | 0 | 674 | 696 | 96.84 | 3.16 |
|  | **Total** | 1247 | 2070 | 4091 | 674 | 8082 |  |  |
|  | **Specificity (%)** | 99.93 | 99.60 | 99.82 | 100.00 |  | **Overall Accuracy (%)** | **99.55** |
|  | **FPR (%)** | 0.07 | 0.40 | 0.18 | 0.00 |  |  |  |
|  | | | | | | | | |
|  | | **Predicted Results (#pixels)** | | | | |  | |
| **Patient ID** | **Ground Truth Data (#pixels)** | **Normal Tissue** | **Tumor Tissue** | **Blood Vessel** | **Background** | **Total** | **Sensitivity (%)** | **FNR (%)** |
| **4** | **Normal Tissue** | 1836 | 1 | 5 | 0 | 1842 | 99.67 | 0.33 |
|  | **Tumor Tissue** | 0 | 3655 | 0 | 0 | 3655 | 100.00 | 0.00 |
|  | **Blood Vessel** | 267 | 0 | 1246 | 0 | 1513 | 82.35 | 17.65 |
|  | **Background** | 0 | 0 | 0 | 2625 | 2625 | 100.00 | 0.00 |
|  | **Total** | 2103 | 3656 | 1251 | 2625 | 9635 |  | |
|  | **Specificity (%)** | 96.57 | 99.98 | 99.94 | 100.00 |  | **Overall Accuracy (%)** | **97.17** |
|  | **FPR (%)** | 3.43 | 0.02 | 0.06 | 0.00 |  |  |  |
|  | | | | | | | | |
|  | | **Predicted Results (#pixels)** | | | | |  | |
| **Patient ID** | **Ground Truth Data (#pixels)** | **Normal Tissue** | **Tumor Tissue** | **Blood Vessel** | **Background** | **Total** | **Sensitivity (%)** | **FNR (%)** |
| **5** | **Normal Tissue** | 977 | 0 | 0 | 0 | 977 | 100.00 | 0.00 |
|  | **Tumor Tissue** | 0 | 1221 | 0 | 0 | 1221 | 100.00 | 0.00 |
|  | **Blood Vessel** | 111 | 0 | 727 | 69 | 907 | 80.15 | 19.85 |
|  | **Background** | 0 | 0 | 0 | 2503 | 2503 | 100.00 | 0.00 |
|  | **Total** | 1088 | 1221 | 727 | 2572 | 5608 |  | |
|  | **Specificity (%)** | 97.57 | 100.00 | 100.00 | 97.70 |  | **Overall Accuracy (%)** | **96.79** |
|  | **FPR (%)** | 2.43 | 0.00 | 0.00 | 2.30 |  |  |  |

**S3 Table. Confusion matrix results of the SVM supervised classification with RBF kernel applying the 10-fold cross validation method to each patient.**
