# Supplementary material for: Spatio-spectral classification of hyperspectral images for brain cancer detection during surgical operations
Source: PLoS One. 2018 Mar 19;13(3):e0193721. doi: 10.1371/journal.pone.0193721 (PMC5858847; doi:10.1371/journal.pone.0193721)
Supplement: S4 Table — (DOCX) [file pone.0193721.s004.docx]

|  |  | **Predicted Results (#pixels)** | | | | |  |  |
| --- | --- | --- | --- | --- | --- | --- | --- | --- |
| **Patient ID** | **Ground Truth Data (#pixels)** | **Normal Tissue** | **Tumor Tissue** | **Blood Vessel** | **Background** | **Total** | **Sensitivity (%)** | **FNR (%)** |
| **1** | **Normal Tissue** | 2295 | 0 | 0 | 0 | 2295 | 100.00 | 0.00 |
|  | **Tumor Tissue** | 0 | 1221 | 0 | 0 | 1221 | 100.00 | 0.00 |
|  | **Blood Vessel** | 2 | 623 | 706 | 0 | 1331 | 53.04 | 46.96 |
|  | **Background** | 0 | 0 | 0 | 630 | 630 | 100.00 | 0.00 |
|  | **Total** | 2297 | 1844 | 706 | 630 | 5477 |  | |
|  | **Specificity (%)** | 99.92 | 85.35 | 100.00 | 100.00 |  | **Overall Accuracy (%)** | **88.59** |
|  | **FPR (%)** | 0.08 | 14.65 | 0.00 | 0.00 |  |  |  |
|  | | | | | | | | |
|  | | **Predicted Results (#pixels)** | | | | |  | |
| **Patient ID** | **Ground Truth Data (#pixels)** | **Normal Tissue** | **Tumor Tissue** | **Blood Vessel** | **Background** | **Total** | **Sensitivity (%)** | **FNR (%)** |
| **2** | **Normal Tissue** | 4497 | 0 | 19 | 0 | 4516 | 99.58 | 0.42 |
|  | **Tumor Tissue** | 34 | 704 | 117 | 0 | 855 | 82.34 | 17.66 |
|  | **Blood Vessel** | 7 | 2 | 8688 | 0 | 8697 | 99.90 | 0.10 |
|  | **Background** | 26 | 7 | 0 | 1652 | 1685 | 98.04 | 1.96 |
|  | **Total** | 4564 | 713 | 8824 | 1652 | 15753 |  | |
|  | **Specificity (%)** | 99.40 | 99.94 | 98.05 | 100.00 |  | **Overall Accuracy (%)** | **98.65** |
|  | **FPR (%)** | 0.60 | 0.06 | 1.95 | 0.00 |  |  |  |
|  | | | | | | | | |
|  | | **Predicted Results (#pixels)** | | | | |  | |
| **Patient ID** | **Ground Truth Data (#pixels)** | **Normal Tissue** | **Tumor Tissue** | **Blood Vessel** | **Background** | **Total** | **Sensitivity (%)** | **FNR (%)** |
| **3** | **Normal Tissue** | 1242 | 0 | 9 | 0 | 1251 | 99.28 | 0.72 |
|  | **Tumor Tissue** | 0 | 2046 | 0 | 0 | 2046 | 100.00 | 0.00 |
|  | **Blood Vessel** | 1 | 0 | 4088 | 0 | 4089 | 99.98 | 0.02 |
|  | **Background** | 0 | 24 | 0 | 672 | 696 | 96.55 | 3.45 |
|  | **Total** | 1243 | 2070 | 4097 | 672 | 8082 |  |  |
|  | **Specificity (%)** | 99.99 | 99.60 | 99.77 | 100.00 |  | **Overall Accuracy (%)** | **99.58** |
|  | **FPR (%)** | 0.01 | 0.40 | 0.23 | 0.00 |  |  |  |
|  | | | | | | | | |
|  | | **Predicted Results (#pixels)** | | | | |  | |
| **Patient ID** | **Ground Truth Data (#pixels)** | **Normal Tissue** | **Tumor Tissue** | **Blood Vessel** | **Background** | **Total** | **Sensitivity (%)** | **FNR (%)** |
| **4** | **Normal Tissue** | 1837 | 2 | 3 | 0 | 1842 | 99.73 | 0.27 |
|  | **Tumor Tissue** | 0 | 3655 | 0 | 0 | 3655 | 100.00 | 0.00 |
|  | **Blood Vessel** | 393 | 0 | 1120 | 0 | 1513 | 74.03 | 25.97 |
|  | **Background** | 0 | 0 | 0 | 2625 | 2625 | 100.00 | 0.00 |
|  | **Total** | 2230 | 3657 | 1123 | 2625 | 9635 |  | |
|  | **Specificity (%)** | 94.96 | 99.96 | 99.96 | 100.00 |  | **Overall Accuracy (%)** | **95.87** |
|  | **FPR (%)** | 5.04 | 0.04 | 0.04 | 0.00 |  |  |  |
|  | | | | | | | | |
|  | | **Predicted Results (#pixels)** | | | | |  | |
| **Patient ID** | **Ground Truth Data (#pixels)** | **Normal Tissue** | **Tumor Tissue** | **Blood Vessel** | **Background** | **Total** | **Sensitivity (%)** | **FNR (%)** |
| **5** | **Normal Tissue** | 977 | 0 | 0 | 0 | 977 | 100.00 | 0.00 |
|  | **Tumor Tissue** | 0 | 1221 | 0 | 0 | 1221 | 100.00 | 0.00 |
|  | **Blood Vessel** | 111 | 0 | 727 | 69 | 907 | 80.15 | 19.85 |
|  | **Background** | 0 | 0 | 0 | 2503 | 2503 | 100.00 | 0.00 |
|  | **Total** | 1088 | 1221 | 727 | 2572 | 5608 |  | |
|  | **Specificity (%)** | 97.57 | 100.00 | 100.00 | 97.70 |  | **Overall Accuracy (%)** | **96.79** |
|  | **FPR (%)** | 2.43 | 0.00 | 0.00 | 2.30 |  |  |  |

**S4 Table. Confusion matrix results of the SVM supervised classification with sigmoid kernel applying the 10-fold cross validation method to each patient.**
